# The Circles of Resilience model: exploring how social and structural conditions foster child resilience in the context of ACEs

**DOI:** 10.3389/fpubh.2026.1802488

**Published:** 2026-06-22

**Authors:** Clare Viglione, Alec Terrana, Tsung-Chin Wu, Xin M. Tu, Elva M. Arredondo, Noe C. Crespo, Pradeep Gidwani, Job Godino, Kyung E. Rhee, Renée Boynton-Jarrett, Borsika A. Rabin, Eric Hekler

**Affiliations:** 1 Altman Clinical and Translational Research Institute, School of Medicine, University of California San Diego, San Diego, CA, United States; 2 Herbert Wertheim School of Public Health and Human Longevity Science, University of California San Diego, San Diego, CA, United States; 3 Department of Psychiatry and Behavioral Sciences, University of Washington, Seattle, WA, United States; 4 Division of Biostatistics and Bioinformatics, Herbert Wertheim School of Public Health and Human Longevity Science, University of California San Diego, San Diego, CA, United States; 5 School of Public Health, San Diego State University, San Diego, CA, United States; 6 Institute for Behavioral and Community Health, College of Health and Human Services, San Diego State University, San Diego, CA, United States; 7 American Academy of Pediatrics, California Chapter 3, San Diego, CA, United States; 8 Laura Rodriguez Research Institute Family Health Centers of San Diego, San Diego, CA, United States; 9 Department of Pediatrics, School of Medicine, University of California San Diego, San Diego, CA, United States; 10 Department of Pediatrics, Boston University School of Medicine, Boston, MA, United States

**Keywords:** ACEs, child health, child health policy, child resilience, family systems, health equity, parental stress, resilience

## Abstract

**Introduction:**

Despite strong evidence linking adverse childhood experiences (ACEs) to poor health, current healthcare interventions lack robust empirical support, underscoring the need for a new framework to build child resilience across healthcare, policy, community, and family systems.

**Objectives:**

To introduce and provide preliminary empirical evidence supporting the Circles of Resilience (CoR) model by examining whether social connections and vital conditions buffer pathways linking ACEs, maternal anxiety, and child self-regulation in a Federally Qualified Health Center (FQHC) serving Latino families.

**Methods:**

We conducted a cross-sectional study using parent-reported data collected from 2021–2023 at an FQHC in San Diego, California (*N* = 156 Latino mother–child dyads). Measures included ACEs, maternal anxiety, child self-regulation, and protective factors: (1) social connections and (2) vital conditions (housing, food security, transportation, community resources). Structural equation modeling examined interrelationships among ACEs, maternal anxiety, and child self-regulation, including whether maternal anxiety mediated pathways and whether familial protective factors moderated associations.

**Results:**

Higher ACEs were associated with worse child self-regulation (*β* = 7.44, *p* < 0.001). There was an indirect association between ACEs and child self-regulation via maternal anxiety (indirect *β* = 2.48, *p* = 0.006; ACEs → anxiety *β* = 0.67, *p* = 0.007; anxiety → self-regulation *β* = 3.72, *p* < 0.001). Vital conditions were significantly associated with child self-regulation (*β* = −1.81, SE = 0.92, *p* = 0.049), whereas social connections were not (*β* = 2.08, SE = 1.87, *p* = 0.266). Higher social connections weakened the ACEs–maternal anxiety association (interaction *β* = −0.70, *p* = 0.005), while the buffering effect of vital conditions was not significant (*β* = −0.61, *p* = 0.078).

**Conclusion:**

Findings provide preliminary support for CoR model pathways. Higher ACEs were associated with poorer child self-regulation, partially mediated by maternal anxiety. Vital conditions were directly associated with child self-regulation, whereas parental social connections were not. However, social connections significantly buffered the ACEs–maternal anxiety association, suggesting a protective effect on caregiver stress. The buffering effect of vital conditions was not significant. If replicated longitudinally, findings may clarify distinct mechanisms through which contextual resources shape resilience in children exposed to early adversity.

## Introduction

On August 28, 2024, U.S. Surgeon General released a report highlighting the high levels of stress experienced by parents in the United States and the lack of resources to alleviate it (1). The report emphasized the urgent need to support parents and families to help communities thrive, noting that 33% of parents report feeling stressed compared to 20% of other adults (1, 2). Murthy asserted that societal change is needed to value parenting and implement policies, programs, and individual actions that make raising children easier. Families living in poverty or facing structural inequities are particularly vulnerable, as stress during new parenthood can exacerbate mental health challenges and disrupt secure parent–child attachment (2).

Adverse Childhood Experiences (ACEs) are a broad categorization referring to hardships experienced in early life including abuse (physical, emotional, and sexual), neglect (emotional and physical), household dysfunction (e.g., parental mental health and substance abuse problems), and social hardships like food insecurity (3). In population studies, ACEs are associated with poor health and social outcomes and act in a dose–response manner, such that a greater number of ACEs are associated with increased risks of chronic stress and other deleterious outcomes like obesity, heart disease, and substance abuse (4). Similarly, ACEs have also been found to increase the risk of challenges with executive function (EF), which refer to a set of cognitive processes critical to goal-directed action and self-regulation (SR) (5, 6). The hypothesized intra-individual mechanism for this effect is that ACEs lead to repeated activation of the sympathetic nervous system (i.e., toxic stress), an overgeneralization of the stress response (i.e., responding to neutral stimuli with a stress response), and disruption in prefrontal cortex activity, essential for EF and self-regulatory behaviors (5, 7).

Emerging scholarship has shifted away from viewing ACEs as isolated events toward conceptualizing experiences of adversity and resultant toxic stress as dynamic processes moderated by protective resources (8). Adversity becomes harmful when coping supports are insufficient, while resilience emerges when foundational needs—like safety, belonging, and dignity (8, 9)—are met through relational, social, and structural supports. Effective intervention may require addressing multilevel contexts that foster healthy dynamic attunement between groups, such as families, as the dynamical system whereby stress is either successfully navigated or becomes further amplified. This orientation shifts away from one-time screenings toward approaches that hold healthy family dynamics as the core focus that can be nurtured via broader social resources available and connected within communities.

In this vein, we characterize individual child resilience as emergent from healthy family dynamics, operationalized as a secure parent–child attachment, which is then supported by broader social and environmental-level factors that create the conditions for healthy family dynamics to emerge more or less easily. With this approach, we developed the Circles of Resilience model (CoR model) to emphasize the interdependence of nurturing families’ capacities to attune to one another—regardless of their current social and environmental circumstances—and the critical role of social and environmental conditions that, when increasingly present and interconnected, create the context in which caregiver-child attunement can be more fully realized, thereby fostering resilience to the stressors and events children and families may face. This formulation is in contrast to an orientation that focuses on either the individual or the broader factors, or the even more classical orientation of viewing resilience as purely an individual trait.

The orientation of our CoR model relies on a bedrock of research conducted by previous resilience scientists. In 2013, Zimmerman and colleagues articulated the foundational, *resiliency theory*, detailing how resilience is a dynamic, relational process shaped by promotive factors across multiple ecological levels (10). At the time, this assertion represented a theoretical shift reframing resilience as something that is cultivated through relationships and systems, rather than a trait that someone possesses. Zimmerman et al. (10) urged that resilience is understood through models that capture cumulative and interactive effects of factors across individual, family, and community domains. This orientation also draws upon Ann Masten’s immense body of work, which posits that resilience is a dynamic and multilevel process of positive adaptation in the face of adversity, rooted in developmental science (11). Resilience to adversity arises from the interplay of biological, psychological, social, and cultural processes, with adaptive capacity shaped by protective factors like supportive relationships (11).

Froma Walsh’s family resilience framework conceptualizes resilience not as an individual trait but as a process emerging through family belief systems (e.g., meaning-making and positive outlook), organizational patterns (e.g., flexibility, connectedness, and access to resources), and communication/problem-solving processes (e.g., clarity, emotional expression, and collaborative decision-making), which together shape how families adapt and maintain coherence under adversity (12). Building on this foundation, Hamby, Grych, and Banyard’s Resilience Portfolio Model advances a multidimensional, strengths-based approach that emphasizes the cumulative and synergistic effects of “poly-strengths” across individual, relational, and community domains, framing resilience as the combined availability and use of diverse protective resources rather than a single buffering factor (13). Extending this, the present CoR model moves beyond lists of isolated buffers to a systems-oriented perspective in which interaction is understood as the co-activation and coupling of conditions across individual, relational, and contextual domains that jointly shape resilience. More specifically, in the CoR model, social and structural conditions are theorized to support parents’ and children’s capacity for co-regulation.

Central to this *systems* orientation, is the role of the parent–child co-regulatory relationship as the proximal mechanism through which broader social and structural conditions influence child development. Co-regulation refers to the dynamic, bidirectional process through which caregivers support children in managing emotional and physiological states—providing the scaffolding through which children gradually internalize self-regulatory capacities (14, 15). The idea is that when caregivers are themselves overwhelmed by stress, their capacity to provide consistent, attuned co-regulation is compromised, disrupting the relational conditions children depend on for healthy emotional development. Parental stress and mood disorders—including anxiety, depression, and emotion dysregulation—have been associated with less responsive caregiving, reduced emotional availability, and heightened parent–child conflict, each of which can undermine children’s emerging self-regulation (1, 16–18). Conversely, when parents experience lower levels of stress and higher social support, they are better positioned to engage in the kind of sensitive, predictable caregiving that fosters children’s capacity to regulate emotion and behavior (17, 19). This suggests that the pathway from adversity to child outcomes may be partially routed through parental mental health, and that interventions targeting parental stress—particularly in the context of ACEs—may have cascading benefits for child self-regulation and resilience. These dynamics may be further moderated by the broader social and structural conditions in which families are embedded: families with access to diverse social supports, stable housing, reliable transportation, and economic security may be better buffered against the stress-propagating effects of adversity, creating conditions more conducive to healthy co-regulation dynamics and, by extension, child resilience.

Child self-regulation—the capacity to manage emotions, attention, and thoughts and perform goal-directed behaviors—is widely recognized as a strong outcome indicator for child resilience, representing a child’s ability to cope, adapt to changing circumstances, and engage effectively in social and learning contexts (14, 20). Rather than a fixed trait, self-regulation is increasingly understood as the contextually influenced manifestation of supportive relationships through co-regulation with caregivers, contrasting with earlier views that framed it as a stable characteristic. Self-regulation can be seen as an indicator of the extent to which a child is able to and function and cope effectively with life’s adversities. Evidence also suggests that self-regulation may mediate the association between ACE exposure and shortened telomere length (16, 21), underscoring its role as a pathway through which early adversity influences downstream morbidity.

We developed the Circles of Resilience (CoR) model as a multilevel framework that conceptualizes child resilience (and thereby self-regulation) as emerging from nested, interdependent systems spanning structural conditions, social relationships, and family dynamics. CoR broadly conceptualizes resilience as nested, interdependent factors spanning individual, relational, and societal levels. The model draws on complementary frameworks: *Seven Vital Conditions for Well-Being Framework* (22) provides a structural perspective on the conditions necessary for health and well-being, including safe environments and access to essential resources. Vital Conditions (22), aligns with the outermost circle of the CoR and serves as a primary moderator of interest. Research suggests that safe and supportive neighborhoods buffer the effects of ACEs on child health (23),. There is also some evidence that social connections and social support can buffer the effects of ACEs on mental health (24, 25), but to our knowledge this is not replicated with child self-regulation as an outcome and there is a dearth of research evaluating social factors as buffers in Latino populations.

By investigating the CoR pathways within a Latino population in San Diego, the study provides an initial look on how social and environmental factors may disrupt the effects of ACEs. These findings contribute to a more nuanced understanding of the mechanisms linking early adversity to child well-being and highlight potential protective factors that can be leveraged across healthcare and community contexts to promote resilience. The *Strengthening Families Framework e*mphasizes protective processes within families, such as parental resilience and social connections. The Circle of Security model contributes a relational lens focused on caregiver–child attachment and emotional attunement. Together, these frameworks highlight how structural, relational, and family-level factors interact to shape child development and resilience. The CoR model positions the family as the central context through which broader social and environmental conditions are experienced and translated into developmental outcomes.

This paper pursues two aims. Aim 1 is to propose and articulate the CoR model as an organizing framework for research and practice efforts directed at promoting childhood resilience. Aim 2 is to provide a preliminary cross-sectional empirical test of the model using structural equation modeling. Specifically, we (1) examined the association between child ACEs and child self-regulation; (2) tested whether the family environment (Vital Conditions composite score) (22) and maternal social connections (measured by the Parents Assessment of Protective Factors) (26) moderate the assocation between child ACEs and child self-regulation; and (3) assessed whether maternal anxiety and maternal emotion regulation mediate that same relationship.

## Methods

### Development of the circle of resilience model (aim 1)

The CoR model was developed through an integrative, iterative process combining literature review, theoretical synthesis, and team deliberation. We began by reviewing research on adverse childhood experiences (ACEs), child resilience, and self-regulation, as well as key frameworks for understanding protective factors, family dynamics, and social determinants of health. Insights were drawn from multiple theoretical and applied models, including the Seven Vital Conditions for Well-Being Framework (22) (outer circle), the Strengthening Families Framework (27) (middle circle), the Circle of Security (28, 29) model (inner circle), and foundational resilience theory articulated by Zimmerman (10) and Masten (11), which emphasizes resilience as a dynamic, relational process across ecological levels.

Model development was structured according to Borsboom’s Theory Construction Methodology (TCM) (30), a systematic approach to building psychological and behavioral theories that moves from conceptual identification through formal specification and empirical testing. TCM provides explicit steps for translating observed phenomena into coherent, testable theoretical frameworks. In alignment with Step 1 of TCM—phenomenon identification—we began by defining the outcome: why some children develop adaptive functioning and self-regulation despite exposure to adversity, while others do not. This step required specifying the boundaries of the phenomenon, conceptualizing resilience as a context-dependent process from static trait-based accounts, and identifying the ecological levels (individual, family, community, environment) across which it operates.

Step 2 of TCM—theoretical construct identification—guided our selection of the core constructs to be included in the model. Drawing deductively from established theory, we identified modifiable constructs proximal to child outcomes: caregiver attunement and child self-regulation at the family level, social connectedness and support at the community level, and vital conditions (e.g., housing stability, economic opportunity, belonging) at the environmental level. Interrelationships among constructs were specified using deductive reasoning from existing frameworks, then refined through abductive reasoning—iteratively generating and evaluating hypotheses about how conditions at each ecological level influence those nested within it. The result of this process is a prototheory—in TCM terms, a structured but not yet fully formalized theoretical depiction—which provides a conceptual basis for hypothesis generation, empirical testing, and, particularly once a formal model that can properly represent the theorized complexity of the phenemonon with fidelity (often via computational modeling), ultimately the design of multi-level interventions to promote resilience, consistent with the methodological principles for theory construction outlined by Borsboom (30).

### Testing the circle of resilience model (aim 2)

Guided by the CoR model, this study examines whether specific protective factors buffer the relationship between ACEs and Self-Regulation (SR) [marker of adaptive child resilience (31)], providing initial evidence for subsequent experimental or longitudinal investigations and informing potential intervention approaches. Specifically, this analysis probes how social resources—derived from the Vital Conditions framework (Outer Circle)—and social connections (Middle Circle) may moderate the association between ACEs and SR, and whether parental mood (i.e., maternal anxiety and emotion regulation) serves as a statistical mediator of this relationship.

Self-regulation was selected as the primary outcome measure of individual adaptive resilience in this study because it is a foundational indicator of adaptive functioning and emotional well-being in children (7, 32), especially in the context of adversity. As the ability to manage thoughts, emotions, and behaviors in pursuit of long-term goals, self-regulation is widely recognized as a core capacity that supports academic achievement, social competence, and mental health (14, 20).

### Study design (aim 2)

To generate preliminary cross-sectional empirical testing of the CoR model, we (1) examined the association between child ACEs and child self-regulation; (2) Tested whether the family’s environment (Vital Conditions (22) composite score) and maternal social connections [measured by the Parents Assessment of Protective Factors (26)] statistically moderate the relationship between child ACEs and child self-regulation; and (3) assessed whether maternal anxiety and maternal emotion regulation serve as statistical intermediaries in the relationship between child ACEs and child self-regulation. This cross-sectional analysis examines how parent-reported ACEs experienced by the child (exposure) relate to child self-regulation (outcome), and whether these relationships are moderated by the parent’s familial environment, as measured by a Vital Conditions composite score reflecting the outer circle of the CoR (moderator 1), and by maternal social connections, reflecting the middle circle of CoR (moderator 2). We also examine key statistical mediators, maternal anxiety (mediator 1) and maternal emotion regulation (mediator 2), integrating the parent role into the pathway, thereby representing the center loop of parent–child relationship. Finally, we tested whether Vital Conditions and maternal social connections could statistically moderate these mediated pathways.

**Figure 1:**
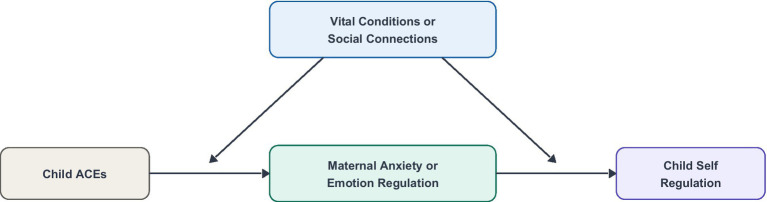
Depicting primary moderators: vital conditions and social connections.

This analysis utilizes existing baseline data from a completed, randomized factorial trial the HEALthy4You (H4Y) Study, which emerged as a partnership between researchers at University of California, San Diego; a Federally Qualified Health Center (FQHC) in San Diego; the San Diego County Childhood Obesity Initiative, a multi-sector coalition addressing children’s health through collective impact; the American Academy of Pediatrics; and the Comité Organizador Latino de City Heights, with funding from the California Institute for the Advancement of Precision Medicine, focusing on addressing ACEs and health. The umbrella study recruited mother–child dyads between 2021 and 2023 from the FQHC for a factorial study evaluating intervention enhancements (i.e., a community health worker to address social needs and a parenting program inspired by evidence-based interventions to promote parenting skills) to the existing 6-month HealthyTogether lifestyle and weight loss program Figure 1.

#### Study sample (aim 2)

The eligibility criteria for the H4Y study were parent participants who had children enrolled in pediatric clinical care. The sample consisted of mothers or female legal guardians who self-identify as Hispanic or Latino (age 18 years or older) and their children (ages 5–11) with obesity (BMI ≥ 85th percentile to 99.9th percentile). Dyad participants consist of one mother (or female legal guardian) and one child. Parents were referred to the study by the child’s primary care provider in 2022 and 2023. Exclusion criteria for the parent study included severe developmental delay in the child or severe mental illness impacting one’s ability to participate in either parent or child.

#### Data collection (aim 2)

Data were collected via telephone or electronic survey at baseline by trained research assistants (approximately 1 h for survey completion) and stored in REDCap, a secure web application for building and managing online surveys and databases. Survey data were uploaded into RStudio (R 3.6.0) for analyses. Survey measures were published in English and Spanish and have been used with Spanish-speaking populations in the United States and included the Whole Child Assessment (WCA) (33), the Behavior Rating Inventory of Executive Function (BRIEF-2) (34), the Generalized Anxiety Disorder (maternal anxiety) (35), and the Parent Assessment of Protective Factors (26). Measures were translated by the instrument publishers and available for completion in Spanish or English. Parents received a gift card for $50 for completing the survey. The survey measures were approved as a component of the UC San Diego IRB review (protocol #802492) and consent was acquired from all parent participants Table 1.

**Table 1:** Study data sources and variables of interest.

Variable type	Domain	Instrument	Measure	Coding
Independent Variable	Adverse childhood experiences	Whole child assessment (WCA) (28)	Total ACEs exposure score (continuous)	0–10 Higher scores = greater adversity
Mediator 1	Maternal anxiety	Generalized anxiety disorder −7 (GAD-7) (30)	Total score (continuous)	0–21 Higher scores = greater anxiety
Mediator 2	Maternal emotion regulation	Parents assessment of protective factors (PAPF); social emotional competence subscore (31)	Subscale average (continuous)	0 = This is not at all like me or what I believe 4 = This is very much like me or what I believe
Moderator 1	Vital conditions	Select items from PRAPARE (30), WCA (28), and PAPF	Total score (continuous)	0–6 Higher scores = greater number of vital conditions represented
Moderator 2	Social connections	Parents assessment of protective factors (PAPF); social connections subscore (31)	Subscale average (continuous)	0 = This is not at all like me or what I believe 4 = This is very much like me or what I believe
Dependent variable	Child self-regulation	Behavior rating inventory of executive function-2 (29)	Global executive total raw score (continuous)	0–100 Higher scores = worse self-regulation

### Study data sources and variables of interest (aim 2)

#### Independent variable: adverse childhood experiences

Adverse Childhood Experiences (ACEs) were measured using the Whole Child Assessment (WCA), a 48-item, parent-report instrument to identify various forms of adversity, including abuse, neglect, household dysfunction, and social hardship (33). The WCA is a California state-approved Individual Health Education Behavioral Assessment tool developed for use during routine well-child visits. It offers age-specific versions in English and Spanish for children from birth to age 20 and has demonstrated concurrent validity with child behavioral outcomes in cross-sectional studies (33, 36). Using WCA, ACEs were operationalized as *household*-level adversities reflecting stressors in the caregiving environment. Total ACE scores were calculated by summing items (0 or 1) reflecting exposures to ACEs resulting in a total continuous score of 0 to 10. ACE scores were derived from ten indicators spanning abuse, family disconnection, parental substance use, and parental justice involvement. Higher scores indicate greater exposure to household-level adversities. Specifically, these included caregiver verbal aggression (e.g., yelling, swearing, or insulting the child), physical and sexual abuse, lack of perceived family support or closeness, caregiver concern about food insecurity, parental separation or divorce, witnessing domestic violence, household substance use, household mental illness or suicidality, and household member incarceration. The items reflect experiences affecting the caregiver and the child, and we conceptualize the measure as capturing the broader household context. As a relatively new instrument, the WCA continues to undergo validation. The tool has demonstrated concurrent and face validity in previous studies (33, 36). Although reliability has not yet been formally established, given its structure comprising simple, close-ended items rather than scales assessing latent domains, it is reasonable to expect a high degree of consistency in responses.

#### Dependent variable: child self-regulation

Child self-regulation was assessed using the Behavior Rating Inventory of Executive Function, Second Edition (BRIEF-2), Parent Form (34). The 63-item measure evaluates behaviors associated with executive function, with parent responses scored on a three-point Likert scale (“Never,” “Sometimes,” “Often”) (29). The BRIEF-2 generates three indices—Behavioral Regulation Index (BRI), Emotional Regulation Index (ERI), and Cognitive Regulation Index (CRI)—which together yield a Global Executive Composite (GEC) score. The GEC sum score, a higher-order composite reflecting overall executive function difficulties, was used as the primary indicator of child self-regulation in all analyses. Self-regulation is the higher-order construct, with distinct executive functions as components that helps individuals manage and control their actions and emotions effectively (7, 20). GEC raw scores range from 30 to 120, with higher scores indicating greater executive function challenges and worse self-regulation. The BRIEF has many studies confirming the reliability and validity of the instrument. The BRIEF-2 (i.e., revised version 2) was developed through a rigorous process including literature review, expert input, large-scale pilot testing, and exploratory factor analysis to ensure content validity and conceptual grounding in executive function theory (34). The revision from the original BRIEF to BRIEF-2 aimed to reduce item burden while maintaining strong psychometric properties, with studies consistently showing good internal consistency and test–retest reliability (37). A Spanish-language version of the BRIEF-2 has also been validated with moderate model fit across the three factors or indices (37). Child self-regulation was used as a marker of child resilience.

#### Statistical mediator 1: maternal anxiety

Parent anxiety symptoms were measured using the Generalized Anxiety Disorder – 7 Item (GAD-7), a widely used clinical screener and validated self-report scale (38). The GAD-7 includes seven items scored from 0 (not at all) to 3 (nearly every day), assessing the frequency of anxiety symptoms over the past 2 weeks. Total scores range from 0 to 21 and were used as a continuous score representing anxiety severity, with higher scores indicating worse anxiety. The GAD-7 has longstanding and established reliability and validity from numerous studies (35, 39).

#### Statistical mediator 2: maternal emotion regulation

The Social Emotional Competence scale from the Parent Assessment of Protective Factors (PAPF) measure assesses a parent’s ability to maintain emotional control and engage in positive parenting behaviors in response to child behaviors (26). The scale consists of 8 items, including: “I help my child learn to manage frustration,” “I stay patient when my child cries,” and “I play with my child when we are together.” Caregivers rate these items on a 5-point scale, where 0 = “This is not at all like me or what I believe” and 4 = “This is very much like me or what I believe.” The subscale score is calculated as the average score of all 8 items, where higher scores indicate greater emotion regulation and associated parenting behaviors. The PAPF demonstrates strong psychometric properties, with content, face, and convergent validity with similar measures well-established. Reliability, assessed via internal consistency, is also high across subscales (26).

#### Statistical moderator 1: vital conditions

The Vital Conditions sum score (VitalConditSum) was developed for this study based on the Vital Conditions for Well-Being Framework, published by the Centers for Disease Control and Prevention and Rippel Foundation (22). The framework outlines seven essential societal conditions that all people need to thrive, including humane housing, meaningful work and wealth, lifelong learning, reliable transportation, basic needs for health and safety, belonging and civic muscle, and a thriving natural environment. We operationalized five of these—housing, education, transportation, work, safety, and health—using survey items aligning with these domains. Belonging/Civic Engagement and Thriving Natural Environment were not included due to the absence of corresponding items in the parent dataset. The sum score includes six indicators of vital conditions: housing stability, child health and access to care, reliable transportation, employment status, educational attainment, and perceived neighborhood safety. Each variable is dichotomized into a binary score (0 or 1) to reflect whether the condition is met. The total VitalConditSum yields a score ranging from 0 to 6, where higher scores representing stronger foundational conditions for health. This composite score also represents the strength of the Outer Circle in the CoR model. Of note, the main independent variable, the ACEs score, includes experiences of adversity that can be viewed as traumatic (e.g., parent addiction, parent severe mental illness, etc.) whereas the Vital Conditions are considered societal resources need to thrive. Of note, the VitalConditSum is a direct composite measure of observable familial conditions rather than a latent psychological construct. Since each item reflects a distinct domain (e.g., housing, transportation, education), the index, traditional psychometric validation and reliability testing is not warranted. Equal weighting was used for transparency and interpretability and is consistent with approaches used in established indices of social conditions (e.g., Area Deprivation Index).

#### Statistical moderator 2: social connections

The Social Connections subscale from the Parent Assessment of Protective Factors (PAPF) measures the extent to which a caregiver feels supported by others and has access to social resources in times of need (26). The scale consists of 9 items, including: “I have someone who will help me get through tough times,” “I have someone who helps me calm down when I get upset,” and “I have someone who can help me calm down if I get frustrated with my child.” Caregivers rate these items on a 5-point scale, where 0 = “This is not at all like me or what I believe” and 4 = “This is very much like me or what I believe.” The subscale score is calculated as the average score of all 9 items, where higher scores indicate greater social connectedness. As mentioned, the PAPF demonstrates strong psychometric properties, with content, face, and convergent validity well-established. Reliability, assessed via internal consistency, is high across subscales (26). The total score also represents components of the Middle Circle in the CoR model.

#### Statistical analysis for aim 2

We first conducted descriptive statistics and generated visualizations for all variables to assess their distributions and evaluate normality. Robust maximum likelihood estimation (MLR) was employed to correct for non-normality and heteroskedasticity (40). All variables were treated as continuous for the purposes of structural equation modeling (SEM) (41). Analyses were restricted to participants with complete data across all variables limiting the enrolled sample from 224 to 156; no imputation was applied for missingness. Models assumed linear relationships among continuous variables and were estimated using a system of linear regressions. All analyses were conducted using the lavaan package in RStudio (Model: 2024.12.1 Build 563).

We followed a sequential SEM modeling approach to examine relationships between ACEs and child behavioral outcomes (i.e., executive function and internalizing symptoms): (1) Direct Path Models: We first estimated total direct effects of ACEs on self-regulation; (2) Mediation: We tested parallel mediation models testing maternal anxiety and maternal emotion regulation as mediators; (3) Moderation: We tested whether Vital Conditions or Social Connections moderated the ACEs ➔ child self-regulation relationship using interaction terms; (4) Moderated Mediation: We added interaction terms (Maternal Anxiety × Vital Conditions and Maternal Anxiety × Social Connections) to explore whether moderators changed the relationship between mediators and child self-regulation; and (5) Mediated Moderation: We examined whether ACEs × Vital Conditions or ACEs × Social Connections predicted mediators, which was associated with child self-regulation (42). To determine the significance of mediation, we assessed the statistical significance of the indirect path by comparing the unstandardized beta coefficients and *p*-values of the indirect versus direct pathways. For moderation, significance was determined by testing the interaction terms between the predictor and the moderator in the model, with significant interactions indicating a moderating effect. Each model is presented with a visual path diagram and associated statistics (unstandardized beta coefficients). Variance inflation factors (VIFs) were calculated to assess multicollinearity, with a threshold of 2 to identify concerns. To control for inflated Type I error in the moderated mediation models, *p*-values were adjusted using the False Discovery Rate (FDR) method.

### Demographic characteristics

We included child gender, parent age, child age category, and perceived “street race” to describe the sample. Child age was categorized (5–6, 6–8, 9–11 years) to reflect developmental stages in middle childhood. We used “street race”—how participants believe others would categorize them based on appearance—rather than only racial/ethnic categories because this measure has been found to better capture socially assigned race and exposure to discrimination in everyday settings (43).

## Results

**Figure 2:**
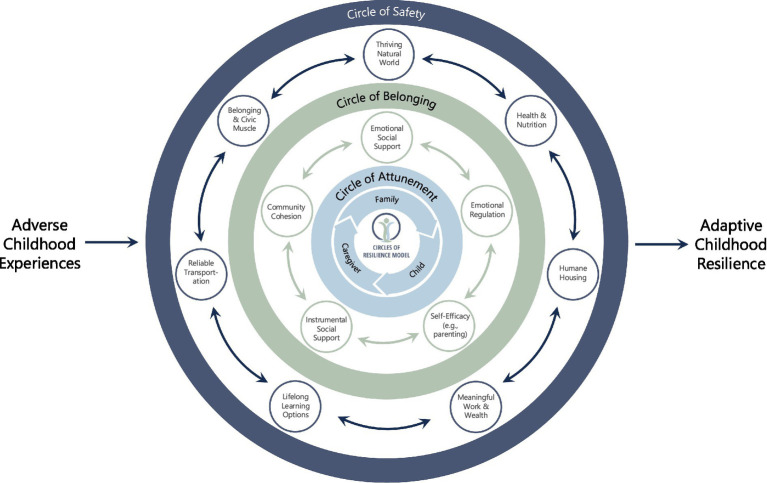
The Circles of Resilience model.

The CoR model (Figure 2) was developed as an early proto-theory (30) (meaning a preliminary narrative and. Visual conceptual model that requires more robust formal operationalization in subsequent work to properly represent the inherent complexity of the phenomenon of interest) to better understand how to address ACEs in real world settings through support of child resilience. The model proposes possible social, familial, and environmental buffers of adversity and frames ‘adaptive childhood resilience’ as a dynamic outcome of interconnected, systemic supports that can be viewed as a nested network (the outer and middle circles) with which families attune to (the inner most circle), which either nurtures or undermines an individual child’s capacity to self-regulate and thus have the requisite ability to respond to any given particular experience of stress. While there may be individual differences in innate ability or temperament to overcome adversity, we hypothesize, based on the CoR, that the amount of variance in self-regulatory capacity is relatively small in relation to these other factors. Beyond that, innate ability or temperament is, definitionally non modifiable and, therefore, is less relevant for guiding the development of interventions to increase children’s resilience.

Put differently, we assume that parents and children, as individuals, and the family as a functional system (inner circle), have varying capacities to adapt to contextual pressures and opportunities. Further, the more supportive their environments/communities (outer circle) and relationships (middle circle) are, the more likely it is that a family and the individuals within the family system will be capable of adapting to any particular life stressors that they may experience. This approach explicitly shifts our understanding of ACEs prevention toward a public health lens by focusing on key modifiable environmental (outer circle) and social (middle circle) factors identified in the literature as contributors to parent–child attachment and well-being (inner circle), and by extension, child resilience. While parents and children can develop capacities to respond to stress regardless of context, these individual-level factors must be understood within the broader framework of environmental and social determinants. The CoR provides an organizing framework to guide future researchers and practitioners interested in cultivating the dynamic processes that nurture resilience of children and families.

The CoR model proposes a holistic, nested, systems model of the family (inner circle) that exists within a broader network-like framework (middle and outer circles) for understanding child resilience and guiding multi-level strategies to enhance child health. Rooted in research on social–emotional health, child development, and protective factors (7, 10, 11, 22, 44), the model conceptualizes resilience across three mutually reinforcing layers, with the outer and middle layers best conceptualized as an interdependent network and the inner most layer as a dynamical system (with the inner system being the key dynamic that is being supported—healthy and resilient families capable of adapting to life stressors). The CoR model offers a way to shift away from the false binary of ACEs as being either something to be resolved at the individual level OR at the social or structural levels and, instead, provides a conceptual map of the ways in which these factors inter-relate, thus providing guidance on shared responsibilities for fostering health, well-being, and resiliency for children and families.

The outermost ‘circle of safety and dignity’ draws on research undergirding the *Seven Vital Conditions for Well-Being Framework* (22) and includes, among other factors, humane housing, meaningful work, access to healthcare, and structural supports necessary for families to thrive. These foundational conditions nurture elements within the second layer, the ‘circle of belonging,’ which focuses on social dimensions including parental social connections with others and parental emotion regulation as critical conditions for stabilizing families and building child resilience, drawing from the *Strengthening Families Framework* (27). The second tier then creates the conditions for a family capable of adapting and attuning to their children, referred to as the “circle of attunement,” which represents the emotional reciprocation between parent and child which allows for a child’s capacity for secure exploration, drawing on principles from the extant *Circle of Security* model (28, 29). Bidirectional arrows between the layers reflect the dynamic and reciprocal nature of resilience-building processes, while also emphasizing each unique layer as necessary but insufficient in isolation to fully understand and nurture resilience. A key notion throughout the model is that emotional security flows through and between the parent to the child, with parents more likely to be capable of providing stability and safety and attuning to their child’s needs when they are supported by social and emotional supports (middle circle) and vital conditions (outer circle), with each factor in each circle being valuable, but with a likely multiplicative benefit the more factors are present for a parent/caregiver. This model provides a conceptual basis for hypothesis generation, population-based research, and multi-level intervention development.

This study integrates multiple complementary frameworks to capture resilience as a multilevel construct. *Seven Vital Conditions for Well-Being Framework* (22) provides a structural perspective on the conditions necessary for health and well-being, while the *Strengthening Families Framework* emphasizes protective processes within the family context. The *Circle of Security* Model contributes a relational lens focused on caregiver-child attachment and emotional attunement. Together, these frameworks inform the CoR model, which conceptualizes child outcomes as emerging from interactions across structural, relational, and family systems levels. The CoR model that we created reflects these more recent theoretical perspectives (Zimmerman; Masten; Walsh; Hamby, Grych, & Banyard) in that no single protective factor (from the middle and outer circles) operates in isolation and that, while each factor may individually contribute, they likely act more effectively the more that are present. Thus, resilience emerges from a dynamic response that can be conceived as a system, which, in this case, is the family. The family, in this model, meets the definition of a system, as articulated in systems science, since the family has multiple elements (i.e., each family member), these elements (family members) interact with one another in predictable ways (described in attachment theory, with our orientation towards the dynamical system described in the circle of security model), and the family has a shared orientation or purpose towards each other, which we define here as attunement and, more broadly capacity to adapt in a healthy way to social and environmental stressors, thus, producing resilience. With this, healthy family dynamics, regardless of context, is the core focus of this model, and those healthy family dynamics are either nurtured or hindered by the availability or lack thereof of broader social and environmental factors.

One can think of a net to understand the ways in which the middle and outer circles are thought to operate. A net requires both a basic number of nodes and that those nodes are interconnected. The more nodes, with more connections, the stronger the net. This is similar to how we conceptualize the ways in which the factors identified in the outer and middle circles function. The more that are present and the more that work well together (e.g., humane housing AND reliable transportation AND meaningful work and wealth, from the outer circle), the stronger the net is, thus, both reducing the likelihood of adverse events AND increasing the likelihood that when adverse events occur, the family is resilient in that they are capable of attuning to (being sensitive to) one another as they navigate the challenge, such that their basic needs of safety, belonging, and dignity can be either maintained or re-established. The outer and middle layer networks provide the supportive (or not) context that impacts the dynamic, relational processes, theorized by Zimmerman (10) in resiliency theory, that are occurring within the inner circle. Thus, resilience is a dynamic, relational process that is shaped by promotive factors across multiple ecological levels.

### AIM 2 results of a preliminary cross-sectional empirical validation of the model

#### Sample description

The analytic sample included 156 caregiver–child dyads, primarily identifying as Hispanic/Latino (98.7%). Children were evenly distributed by gender (52.9% male) and ranged in age from 5–11 years, with nearly half aged 9–11. Caregivers had a mean age of 38.2 years (SD = 6.5). Reported ACE exposure was relatively low on average (M = 1.03, SD = 1.29), though over 60% of children experienced at least one ACE, most commonly verbal abuse, parental divorce, and poverty. Families reported multiple unmet vital conditions (M = 1.01, SD = 1.32), particularly in housing, childcare, transportation, and employment, alongside moderate levels of child self-regulation challenges and generally low caregiver anxiety Tables 2, 3.

**Table 2:** Sample demographic characteristics.

Variable	Category	*N* (%)/Mean (SD)
Child gender (*N* = 156)	Male	82 (52.9)
	Female	73 (47.1)
Child age (*N* = 153)	5 years	20 (15.0)
	6–8 years	47 (35.3)
	9–11 years	66 (49.6)
Parent age (*N* = 153)		38.24 (6.46)
	20–29	23 (15.0)
	30–39	74 (48.4)
	40–49	47 (30.7)
	50–59	9 (5.9)
Hispanic/latino ethnicity (*N* = 154)	Yes	152 (98.7)
	No	2 (1.3)
Street race (*N* = 169)	Mexican/Mexicana/Mexicano	76 (49.4)
	Latina/Hispanic	50 (32.5)
	White/Blanco	16 (10.4)
	American	7 (4.5)
	Asian/Filipina/Filipino	7 (4.5)
	Multiracial	6 (3.9)
	Other	7 (4.5)

Values are presented as mean (SD) or *n* (%). Sample sizes vary due to missing data. Street race refers to the “race you think other Americans who do not know you personally would assume you were based on what you look like.” (43).

**Table 3:** Study variables (*N* = 156).

Variable	Mean (SD)/N (%)
Adverse childhood experiences (ACEs)	1.03 (1.29)
0	58 (37.2)
1	35 (22.4)
2	34 (21.8)
3	15 (9.6)
4	6 (3.8)
5	4 (2.6)
6	3 (1.9)
7	1 (0.6)
Verbal abuse	57 (36.5)
Parental divorce/separation	45 (28.8)
Household poverty	35 (22.4)
Household mental illness	24 (15.4)
Domestic violence	15 (9.6)
Parental incarceration	15 (9.6)
Household substance use	11 (7.1)
Lack of social support	10 (6.4)
Physical abuse	3 (1.9)
Sexual abuse	2 (1.3)
Vital conditions (composite)	1.01 (1.32)
Housing	135 (86.5)
Childcare	92 (59.0)
Work	84 (53.8)
Transportation	83 (53.2)
Safety	80 (51.3)
Education	62 (39.7)
Social connections (PAPF)	3.41 (0.77)
Parent anxiety (GAD-7)	1.85 (3.03)
Parent emotion regulation (PAPF)	3.45 (0.62)
Child self-regulation (BRIEF-2)	85.03 (23.05)

Values are presented as mean (SD) or *n* (%). Sample is reduced to complete analytical dataset. ACEs, Adverse childhood experiences. Vital conditions reflect a summed index across domains of housing, childcare, transportation, work, education, and safety. BRIEF-2, Behavior Rating Inventory of Executive Function, Second Edition. GAD-7, Generalized Anxiety Disorder-7 scale; PAPF, Parenting assessment of protective factors subscale.

#### Main findings

##### Model 1: total effect model (base model)

*Hypothesis 1*: Higher exposure to ACEs for the child will be statistically associated with poorer child self-regulation.

**Figure 3:**
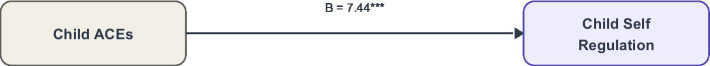
Total effect model (direct path).

Findings: As hypothesized, higher levels of ACEs was found to be statistically associated with poorer child self-regulation, indicated by significantly higher scores on the Behavior Rating Inventory of Executive Function (*β* = 7.44, SE = 1.75, *p* < 0.001). The residual variance in the Behavior Rating Inventory of Executive Function was high (435.68, SE = 56.80, *p* < 0.001), suggesting considerable unexplained variability in the model. Specifically, every one-unit increase in ACEs was associated with 7.44 point increase in child self-regulation scores, reflecting worse executive function Figure 3.

#### Mediation analyses

##### Model 2: parallel mediation model

*Hypothesis 2*: The relationship between higher ACEs and poorer child self-regulation will be statistically mediated by higher maternal anxiety and lower maternal emotion regulation within a cross-sectional dataset.

**Figure 4:**
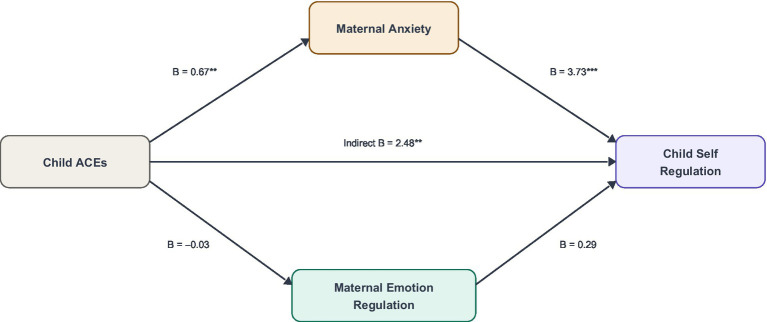
Parallel mediation model.

Findings: Child ACEs were significantly associated with higher maternal anxiety (*β* = 0.67, SE = 0.25, *p* = 0.007), and maternal anxiety was significantly associated with poorer child self-regulation (*β* = 3.73, SE = 0.63, *p* < 0.001). ACEs were not significantly associated with maternal emotion regulation (*β* = −0.03, SE = 0.04, *p* = 0.386), and maternal emotion regulation was not associated with child self-regulation (*β* = 0.29, SE = 2.19, *p* = 0.894). The total indirect association between ACEs and child self-regulation remained significant (*β* = 2.48, SE = 0.91, *p* = 0.006), which seems to have been staistically mediated via maternal anxiety. Every one-unit increase in ACEs was associated with 0.67 unit increases in maternal anxiety, which in turn was associated with a 2.48-point increase in child self-regulation scores. There were no concerns about multicollinearity (variance inflation factor < 2), Figure 4.

##### Model 3: single mediator model (parent anxiety only)

*Hypothesis 3*: Maternal anxiety will partially stastically mediate the relationship between ACEs and child self-regulation.

**Figure 5:**
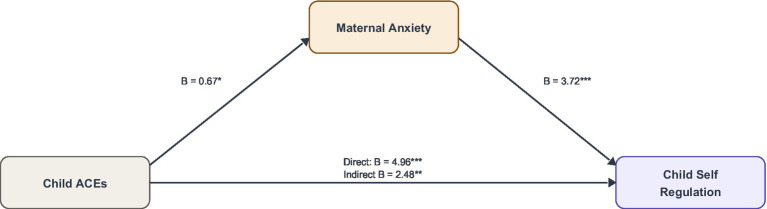
Single mediator path diagram.

*Findings*: The reduced model confirmed a significant indirect statistical mediational relationship between ACEs and child self-regulation through maternal anxiety (*β* = 2.48, SE = 0.91, *p* = 0.006), as well as a significant direct effect (*β* = 4.96, SE = 1.44, *p* = 0.001), supporting *partial* statistical mediation in this cross-sectional analysis. ACEs was significantly associated with maternal anxiety (*β* = 0.67, SE = 0.25, *p* = 0.007), which in turn was statistically associated with poorer child self-regulation (*β* = 3.72, SE = 0.61, *p* < 0.001). Specifically, every one-unit increase in ACEs was associated with a 0.67 maternal anxiety units, and this increase in anxiety was associated with a 3.72-point increase in child self-regulation scores (i.e., worse child self-regulation), Figure 5.

##### Model 4: mediated moderation model (moderation of the ACEs-anxiety relationship)

*Hypothesis 4*: The buffering effects of social connection or vital conditions will be associated with a weaker relationship between ACEs and child self-regulation *via an* attenuating statistical mediation of maternal anxiety. Specifically, the interactions between ACEs and moderators (social connection or vital conditions) will be associated with reduced maternal anxiety, which in turn will be associated with higher child self-regulation.

**Figure 6:**
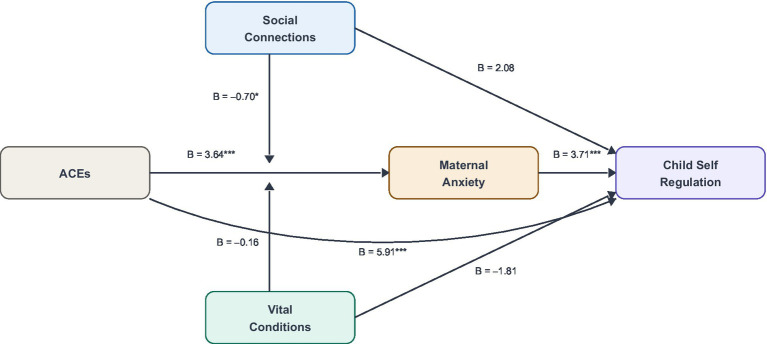
Mediated moderation model (moderation of the ACEs-anxiety relationship).

*Findings*: ACEs were positively associated with both maternal anxiety (*β* = 3.64, SE = 0.98, *p* = 0.001) and child self-regulation (*β* = 5.91, SE = 1.54, *p* < 0.001). Vital conditions were not significantly associated with child self-regulation (*β* = −1.81, SE = 0.92, *p* = 0.065), whereas social connection was not (*β* = 2.08, SE = 1.87, *p* = 0.266). Neither vital conditions (*β* = −0.20, SE = 0.12, *p* = 0.116) nor social connections (*β* = −0.18, SE = 0.49, *p* = 0.708) demonstrated significant main effects on maternal anxiety. However, the interaction between ACEs and social connections was significantly associated with maternal anxiety (*β* = −0.70, SE = 0.25, *p* = 0.013), suggesting that social connection may buffer the association between ACE exposure and maternal anxiety. The interaction between ACEs and vital conditions was not significant (*β* = −0.17, SE = 0.09, *p* = 0.113). Maternal anxiety was significantly and positively associated with child self-regulation (*β* = 3.71, SE = 0.62, *p* < 0.001). Consistent with this pattern, a significant indirect effect was observed for the interaction between ACEs and social connections through maternal anxiety (*β* = −2.61, SE = 0.97, *p* = 0.014), indicating that the buffering effect of social connections on ACE-related anxiety extended to child self-regulation outcomes. In contrast, the indirect effect through the interaction between ACEs and vital conditions was not significant (*β* = −0.61, SE = 0.35, *p* = 0.078), Figure 6.

##### Model 5: moderated mediation model (moderation of the maternal anxiety-child self-regulation relationship)

*Hypothesis 5*: Stronger vital conditions or greater social connectedness will statistically moderate the relationship between maternal anxiety and child self-regulation, thereby weakening the indirect statistical relationships between ACEs and child self-regulation.

**Figure 7:**
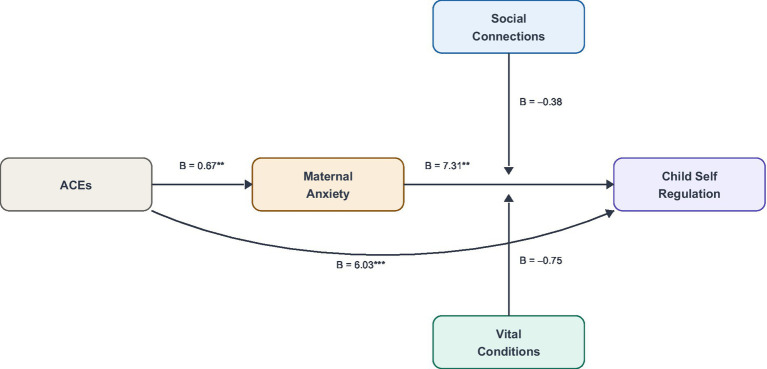
Moderated mediation model (moderation of the maternal anxiety-child self-regulation relationship).

*Findings*: ACEs were positively associated with maternal anxiety (*β* = 0.67, SE = 0.25, *z* = 2.69, *p* = 0.007). When interaction terms were included, maternal anxiety had a significant positive relationship with child self-regulation (*β* = 7.31, SE = 2.16, z = 3.38, *p* = 0.003), and ACEs also retained a significant direct association with child self-regulation (*β* = 6.03, SE = 1.60, *z* = 3.78, *p* = 0.001). Neither vital conditions nor social connections significantly moderated the relationship between maternal anxiety and child self-regulation (*β* = −0.75, SE = 1.07, *z* = −0.70, *p* = 0.485; and *β* = 2.14, SE = 1.81, *z* = 1.19, *p* = 0.354, respectively). The ACEs × Vital Conditions interaction term approached but did not reach significance (bd1: *β* = −0.75, SE = 0.37, *z* = −2.05, *p* = 0.082), and the ACEs × Social Connections interaction was non-significant (bd2: *β* = −0.38, SE = 0.48, z = −0.80, *p* = 0.485). The total indirect effect through the ACEs × Vital Conditions interaction was not significant (*β* = −0.50, SE = 0.32, z = −1.58, *p* = 0.228), nor was the indirect effect through the ACEs × Social Connections interaction (*β* = −0.25, SE = 0.34, *z* = −0.74, *p* = 0.458), Figure 7.

#### Summary of models

Results from five models revealed direct and indirect cross-sectional statistical associations. Model 1 confirmed a significant association between ACEs and child self-regulation (*β* = 7.44, SE = 1.75, *p* < 0.001). In Model 2, maternal anxiety partially mediated this association; ACEs were associated with higher maternal anxiety (*β* = 0.67, SE = 0.25, *p* = 0.007), which in turn was associated with poorer child self-regulation (*β* = 3.73, SE = 0.63, *p* < 0.001), with a significant total indirect effect (*β* = 2.48, SE = 0.91, *p* = 0.006). Maternal emotion regulation was not a significant statistical mediator (path to child self-regulation: *β* = 0.29, SE = 2.19, *p* = 0.894), nor were ACEs significantly associated with maternal emotion regulation (*β* = −0.03, SE = 0.04, *p* = 0.386). Model 3, a reduced single-mediator model, confirmed partial mediation through maternal anxiety, with a significant indirect effect (*β* = 2.48, SE = 0.91, *p* = 0.006) and a remaining significant direct association between ACEs and child self-regulation (*β* = 4.96, SE = 1.44, *p* = 0.001).

Model 4 examined whether vital conditions or social connectedness moderated the ACEs–maternal anxiety association. Social connections significantly moderated this association (*β* = −0.70, SE = 0.25, *p* = 0.013), with a significant corresponding indirect effect on child self-regulation (*β* = −2.61, SE = 0.97, *p* = 0.014), suggesting stronger social ties were statistically linked to a weaker ACEs–anxiety association. Vital conditions did not significantly moderate this pathway (*β* = −0.61, SE = 0.35, *p* = 0.078). Model 5 examined whether vital conditions or social connectedness moderated the maternal anxiety–child self-regulation association. Neither moderator reached significance (vital conditions: *β* = −0.75, SE = 1.07, *p* = 0.485; social connections: *β* = 2.14, SE = 1.81, *p* = 0.354), nor did either indirect effect (ACEs × Vital Conditions: *β* = −0.50, SE = 0.32, *p* = 0.228; ACEs × Social Connections: *β* = −0.25, SE = 0.34, *p* = 0.458).

## Discussion

Our primary contributions of this paper are two-fold: 1) the introduction of the Circles of Resilience (CoR) Model as an approach for guiding researchers and practitioners interested in contributing towards improved childhood resilience to toxic stressors, including ACEs; 2) a preliminary and cross-sectional empirical test of the CoR model.

The CoR model represents a shift in how we conceptualize and address childhood adversity and resilience. The CoR model builds on Bronfenbrenner’s socioecological framework (45) and resilience literature (Zimmerman; Masten; Walsh; Hamby, Grych, and Banyard) (10–13) by identifying not only the multiple levels of protective factors, but *where and how* these factors influence the adversity-to-outcome pathway within families and communities. This model reorients ACEs prevention toward a public health lens by emphasizing modifiable social factors that nurture families’ adaptive capacity, rather than focusing solely on individual-level interventions or non-modifiable innate or temperamental factors. Critically, the model conceptualizes the outer and middle circles as an interconnected network—akin to a net that becomes stronger with more nodes and connections—that provides the supportive context enabling the dynamic processes within the inner circle. With this, we hypothesize that a public health practitioner could have increased capacity to identify key system leverage points to organize actions around to affect the outer circle to strengthen the overall network of factors that promote safety and dignity within a community (e.g., policies to strengthen job security), as well as a secondary focus on the middle layer (e.g., mental health interventions for parents) (46). In contrast, a care provider could primarily orient towards the innermost circle to support parents with increasing their capacity to attune to their child’s needs within whichever contexts they find themselves, with the middle layer as their secondary focus and, by extension, the place where public health and medicine can collaborate effectively. By moving beyond the binary of individual versus structural interventions, the CoR model offers practitioners and researchers a conceptual map for understanding how factors across ecological levels interact to promote child resilience, thereby guiding the development of multi-level, multi-sector strategies to both buffer adversity and cultivate the conditions necessary for families to thrive.

This study provides a first look at pathways proposed in the CoR model, exploring how ACEs, parental mood, and child behavior interrelate and which factors (vital conditions and social connections) might moderate the associations between ACEs and child self-regulation (a proxy for adaptive child resilience). Findings contribute to the growing body of evidence associating ACEs with challenges in self-regulation, with maternal anxiety emerging as a statistically significant mediator. ACEs were associated with elevated maternal anxiety which in turn was associated with poorer child self-regulation, with a significant total indirect effect.

While purely cross-sectional and no causal directionality can be inferred, preliminary results are suggestive that maternal anxiety might serve as an intermediary linking ACEs to child self-regulation and adaptive behavior. Thus, a focus on maternal anxiety or stress could be valuable to support the hypothesized core circle of the CoR, the ‘Circle of Attunement,’ reflecting maternal-child emotional reciprocation, in accordance with the extant Circle of Security framework (28). This also aligns with findings in pre-school students linking ACEs to a range of executive function deficits through maternal stress (47, 48). Per the Circle of Security framework, ‘Being-With’ one’s own child is about being present and listening, which helps children learn that feelings are essential and deserve full availability (28). Parental stress and anxiety might challenge a parent’s ability to be fully present and available, interrupting their ability to be attuned and sensitive to support emotion regulation in their child.

This study also explored the potential roles of two key components of the CoR model: vital conditions and social connections. Though longitudinal work is needed to establish temporal precedence, preliminary patterns suggest these factors may operate through distinct pathways. Social connections appeared more relevant to the upstream segment of the adversity-to-outcome pathway: the ACEs × social connection interaction was significantly associated with maternal anxiety, which in turn produced a significant indirect association with child self-regulation. In contrast, vital conditions showed a significant direct association with child self-regulation, but did not significantly moderate the association between ACEs and maternal anxiety, nor was there evidence of an indirect pathway through maternal anxiety.

These findings do not suggest that vital conditions buffer the association between ACEs and outcomes; rather, they appear to function as a correlate of child self-regulation independent of ACE-related pathways examined here. This distinction is noteworthy. Rather than operating through parental mental health, vital conditions may influence child self-regulation through more proximal mechanisms—such as access to safe environments—that do not require maternal anxiety as an intermediary. Taken together, these patterns suggest that relational supports may be particularly relevant for protecting parental mental health under conditions of adversity, whereas material and environmental supports may influence child outcomes through routes that are more direct. If replicated in larger and longitudinal samples, this distinction could carry implications for intervention design, suggesting that different categories of factors may be most relevant at distinct points along the adversity–stress–development pathway rather than functioning interchangeably.

Findings also suggests that buffering effects were significant for social connectedness specifically when maternal anxiety was included as a mediator, reinforcing the notion that social supports may be particularly relevant for parental mental health, which in turn may be associated with more adaptive child behavior. Put simply, a supported parent may be better positioned to support a child—but the routes through which that support reaches the child may differ depending on whether it is relational or material in nature. These findings are consistent with existing literature suggesting that social support is associated with maternal mental health, although to our knowledge no studies have examined this in relation to child self-regulation (25, 49).

This study represents a first step in studying the Circles of Resilience model within Borsboom et al.’s Theory Construction Methodology (30), which conceptualizes theory development as five iterative steps: identifying empirical phenomena, developing a prototheory, formalizing theory and phenomena, checking explanatory adequacy, and evaluating theory. The present study is positioned within the first two steps: we identified empirical phenomena and developed an initial prototheory (step 1), then tested model pathways using frequentist structural equation modeling (step 2). Our findings offer preliminary associative support for select proposed pathways. At the center of the model, the Circle of Attunement conceptualizes the family dynamic—particularly the parent’s emotional state—as directly linked to child functioning. The role of parental anxiety in the association between ACEs and child self-regulation highlights the potential relevance of emotional attunement within this core circle. Preliminary support also emerged for the model’s proposition that protective factors across ecological levels may operate through distinct mechanisms: social connections appeared relevant primarily through their association with parental mental health, whereas vital conditions showed a more direct association with child self-regulation independent of parental anxiety.

Limitations should be acknowledged. First, given the cross-sectional design, findings are associative and hypothesis-generating, and causal inferences cannot be made; alternative directional relationships (e.g., lower maternal anxiety being associated with greater social connectedness) are also plausible. Second, the relatively modest sample size may have limited power to detect small-to-moderate effects, particularly for interaction and indirect pathways, increasing the risk of Type II error; replication in larger, independent samples is needed. Relatedly, restricting analyses to complete cases (*N* = 156) may introduce selection bias, as the inability to evaluate patterns of missingness across all variables precludes assessing whether data were missing completely at random. Future analyses incorporating sensitivity approaches to missing data would strengthen confidence in these findings. Third, reliance on self-report measures may introduce reporting bias and shared method variance, particularly for sensitive constructs such as adverse childhood experiences and mental health, including potential underreporting. Fourth, models were estimated without adjustment for demographic covariates (e.g., child age, socioeconomic status, maternal education, and household structure). While this decision was intended to avoid overadjustment for variables closely tied to structural conditions and exposure pathways, it increases susceptibility to residual confounding, and observed associations may partly reflect unmeasured contextual differences. Finally, the sample was restricted to children with obesity, a multifactorial condition shaped by biological, behavioral, and social determinants, limiting generalizability and raising the possibility of condition-related confounding or effect modification.

More broadly, frequentist structural equation modeling in a cross-sectional nomothetic dataset assumes linearity and cannot capture the dynamic, reciprocal, and time-sensitive nature of the constructs theorized by the CoR model. These results are explicitly population-level estimates that do not necessarily translate to individual cases, as the assumption of ergodicity is unlikely to be met. For these reasons, the CoR is explicitly labeled a *prototheory*: while aligned with standards of rigor common in public health and psychology, a purely visual and textual description lacks the precision needed to represent the complexity of these phenomena. Advancing to step 3 of the Theory Construction Methodology will require formal computational approaches—such as network analysis, system dynamics modeling, or agent-based modeling—capable of representing the non-linear, feedback-driven, and emergent interactions that remain underspecified in the current model.

Overall, these findings should be considered preliminary and hypothesis-generating, offering a foundation for longitudinal, mixed-methods, and multi-level studies that more fully examine these interrelated processes in real-world contexts. In terms of future experimental approaches, research could involve embedded pragmatic trials, randomized micro-interventions, or adaptive experimental designs (e.g., optimization trials as used in the Multiphase Optimization Strategy) that test specific factors and pathways in the system. For example, interventions targeting maternal anxiety could be experimentally manipulated to test effects on parent–child relational health or child executive function. These methods would allow for empirical testing of pathways while maintaining sensitivity to individual variation and contextual complexity. A key next step, justified by the results of this frequentist statistical approach, is the creation of a formalized and explanatory theory structure [step 3 of Borsboom’s Theory Construction Methodology (30)] that can better account for the inherent level of complexity of the phenomena of interest.

Future intervention strategies could consider how maternal social connections seem important for protecting maternal mental health under conditions of adversity, whereas vital conditions may play a role in supporting child self-regulation. If confirmed in larger studies, these findings may hold relevance for healthcare researchers and policymakers. There is a disconnect between increasingly widespread screening for ACEs and the availability of evidence-based guidelines and approaches to address the effects of childhood trauma and build child resilience. Despite the paucity of interventions for ACEs, healthcare policies like *ACEs Aware* continue to incentivize primary care providers to universally screen for ACEs in practice (50). Screening initiatives like California’s *ACEs Aware* aim to promote trauma-informed care, yet many healthcare systems lack clear pathways for follow-up support and integrative treatments (50, 51). This disconnect raises concerns about the ethical implications of identifying adverse events without offering effective, personalized strategies for families. This research study represents a first step on the quest to identify novel targets for interventions, multi-sector collaborations, and policies to address early adversity and build child resilience. Initiatives such as California’s ACEs Aware program incentivizes providers to screen for ACEs, yet evidence-informed approaches to address identified adversity remain limited.

There is a need for more targeted research examining interventions that address adversity with a well-formulated understanding of resiliency and how to promote it. Although universal ACEs screening is gaining traction, its impact remains limited and it often lacks the nuanced interpretation needed for guiding effective follow-up and action (3, 8). Most interventions targeting ACEs have centered on psychological treatment, with cognitive-behavioral therapy shown to reduce negative effects of certain ACEs like abuse (52, 53). However, evidence for other approaches, such as parenting interventions, remains mixed, with promising findings but inconsistent outcomes (52). Further, despite growing interest in resilience and child mental health, there is a notable gap in research examining how protective factors across ecological levels shape the development of self-regulation. Self-regulation is widely recognized as a foundational component of individual resilience. Individuals with stronger emotional and behavioral self-regulatory capacities are generally better able to adapt to changing circumstances, recover from stress, and maintain emotional stability in the face of diverse challenges (32). In our model, we understand child self-regulation as an emergent individual capacity shaped through ongoing interactions with supportive social environments and attuned caregivers.

Much of the existing literature has also focused on predominantly White or heterogeneous samples, often overlooking the unique cultural, structural, and contextual experiences that shape developmental outcomes in historically underserved groups. Addressing this gap is essential for developing equitable, population-specific strategies to promote resilience and reduce disparities in early childhood. Overall, substantial gaps remain both in creating a coherent theoretical model and in the evidence base, as current research tends to separate individual psychological impacts from the social and contextual pathways. The challenge is how to address both the individual-level effects and the broader relational and life conditions together rigorously and systematically.

Our research highlights the importance of looking at intervention targets beyond individual treatment to include family and dyadic-focused and ecological approaches aligning with the CoR model. Moreover, the observed interrelations suggest that interventions may need to be tailored based on a family’s unique configuration of risk and protective factors. In a recent commentary on the challenges for ACEs screening, Danese et al. assert that individual risk modelling approaches could be adopted to improve the accuracy of ACEs screening and guide intervention selection, and promising interventions need be tested to ensure that vulnerable individuals detected through ACEs screening receive effective support (54). Precision health strategies, rooted in a complex understanding of how vital conditions, social connections, and parental mental health interact, could help close this treatment gap. Sustainable progress in addressing early adversity and building resilience will likely require not only individualized supports for parents and children, but systemic efforts to foster environments and “vital conditions” that promote healthy emotional functioning. With that said, defining the “right” support for each family, based on the current results, necessitates more formalized modeling to study, understand, and unpack these dynamics, to, ultimately, support the creation of precision treatment regimen recommendations.

## Conclusion

The Circles of Resilience (CoR) model offers an action-oriented framework to bridge the gap between widespread ACEs screening and the limited availability of effective interventions. By framing child resilience as emerging from the interplay of vital conditions, social connections, and parent–child attunement, CoR provides a structure to guide researchers, practitioners, and policymakers supporting families facing adversity. Preliminary findings align with the model’s propositions, suggesting that parental emotional health may link adversity to child behavioral outcomes, while social supports may buffer these effects. Future work should refine the model using computational and complex systems approaches, test proposed mechanisms longitudinally, and advance precision health strategies that tailor supports to families’ unique risk and protective profiles. Ultimately, building child resilience will likely benefit from moving beyond the individual “screen-to-treat” paradigms toward multi-level, multi-sector approaches that simultaneously strengthen families’ emotional and relational capacities while fostering the broader conditions necessary for them to thrive.

## Data Availability

The data analyzed in this study is subject to the following licenses/restrictions: Datasets available upon request. Requests to access these datasets should be directed to cviglione@ucsd.edu.
